# Evaluation of Phototrophic Stream Biofilms Under Stress: Comparing Traditional and Novel Ecotoxicological Endpoints After Exposure to Diuron

**DOI:** 10.3389/fmicb.2018.02974

**Published:** 2018-11-29

**Authors:** Linn Sgier, Renata Behra, René Schönenberger, Alexandra Kroll, Anze Zupanic

**Affiliations:** Department of Environmental Toxicology, Eawag – Swiss Federal Institute of Aquatic Science and Technology, Dübendorf, Switzerland

**Keywords:** Ecotoxicology, diuron, stream biofilms, flow cytometry, community structure, photosynthetic efficiency, EPS

## Abstract

Stream biofilms have been shown to be among the most sensitive indicators of environmental stress in aquatic ecosystems and several endpoints have been developed to measure biofilm adverse effects caused by environmental stressors. Here, we compare the effects of long-term exposure of stream biofilms to diuron, a commonly used herbicide, on several traditional ecotoxicological endpoints (biomass growth, photosynthetic efficiency, chlorophyll-a content, and taxonomic composition), with the effects measured by recently developed methods [community structure assessed by flow cytometry (FC-CS) and measurement of extracellular polymeric substances (EPS)]. Biofilms grown from local stream water in recirculating microcosms were exposed to a constant concentration of 20 μg/L diuron over a period of 3 weeks. During the experiment, we observed temporal variation in photosynthetic efficiency, biomass, cell size, presence of decaying cells and in the EPS protein fraction. While biomass growth, photosynthetic efficiency, and chlorophyll-a content were treatment independent, the effects of diuron were detectable with both FC and EPS measurements. This demonstrates that, at least for our experimental setup, a combination of different ecotoxicological endpoints can be important for evaluating biofilm environmental stress and suggests that the more recent ecotoxicological endpoints (FC-CS, EPS protein content and humic substances) can be a useful addition for stream biofilm ecotoxicological assessment.

## Introduction

The concentrations of contaminants in surface waters vary substantially in space and time. Fluxes of pollutants, such as pesticides, can change rapidly and lead to increase in exposure of organisms living in aquatic ecosystems (Wauchope, 1978; Finizio et al., 2005; Blanchoud et al., 2007; Rabiet et al., 2010; Moschet et al., 2014). Among the most strongly affected are stream biofilms, often referred to as periphyton (Azim, 2005) – taxonomically diverse and dynamic communities of phototrophic and heterotrophic microorganisms. They play an important role in stream ecosystems by providing food for higher trophic levels, producing oxygen, changing near-bed hydraulics and providing habitat for protists and invertebrates (Lamberti, 1996; Nikora et al., 1997). Due to their ecological importance, assessing the effects of stressors to stream biofilms has been the subject of many studies, mainly focusing on functional endpoints, such as growth of biomass and photosynthetic efficiency, and on structural endpoints, such as the biofilm taxonomic composition and biodiversity (Dorigo et al., 2007; Ricart et al., 2009; Pesce et al., 2010a; Tlili et al., 2011).

A limitation of the traditional functional endpoints is that they are affected by structural compensation: it is possible for a functional endpoint to remain unchanged, even while the species composition of the biofilm is changing. A limitation of structural endpoints is that their measurement is expensive and/or time consuming, and that no functional information is gained from it. Also, most traditional methods concentrate on the cellular part of the biofilm and ignore the intercellular space that binds the biofilm together. Recently, new biofilm characterization methods have been developed that can simultaneously measure both functional and structural features. Members of our laboratory have, in particular, focused on characterization of freshwater biofilms based on flow cytometry derived community structure (FC-CS) (Sgier et al., 2016, 2018) and liquid chromatography coupled to organic carbon and nitrogen detection (LC-OCD-OND) for characterization of extracellular (polymeric) substances (EPS) present in biofilms (Stewart et al., 2013; Kroll et al., 2014). Both methods measure multiple features of biofilms. FC quantifies fluorescence properties and light scattering properties, related to size and granularity of individual particles in the biofilm, which are then used to cluster the particles into subpopulations. The changes in the number of subpopulations and their size in time reflect changes in the community structure. The community structure, which depends on the biofilm species composition and phenotypic variability (Staley et al., 2015), has been shown to be both affected by stress as well as to be a determining factor in stress resistance (Wahl et al., 2012). LC-OCD-OND can be used to quantify a large size range of organic molecules, including polysaccharides, proteins and acids in the EPS. Changes in the EPS of stream biofilms have been shown to be important for nutrient cycling, biofilm stability, and the interaction with pollutants (Flemming and Wingender, 2010; Stewart et al., 2013; Kroll et al., 2016). Interaction with silver nanoparticles, for example, is mediated by carboxylic groups in stream biofilm EPS (Sambalova et al., 2018). Importantly, there have been only a few studies yet that have compared the effects of environmental stress on stream biofilms on these different endpoints using the same samples, and none of them focused specifically on endpoint sensitivity. Therefore, it is not clear whether any of these endpoints is more sensitive and whether their combined use can provide a more comprehensive understanding of the effects of environmental stress on the biofilms.

In this study, our objective was to compare the effects of long-term chemical exposure on several traditional and more recently developed ecotoxicological biofilms endpoints. As an example chemical, we used DCMU (trade name diuron), a commonly used herbicide (Balsiger et al., 2004; Wittmer et al., 2011; Moschet et al., 2014) that targets the electron transport in photosystem II (PSII) (Vanrensen, 1989) and thereby the phototrophic organisms present in the biofilm. Our choice of stressor is also reflected in our selection of endpoints, which are centered on phototrophic organisms: photosynthetic efficiency, chlorophyll-a content, taxonomy by light microscopy (LM), FC-CS, while both, phototrophic and heterotrophic organisms contribute to the endpoints biomass growth and EPS composition. Since neither FC nor EPS measurements have been used before in diuron studies, we hypothesized using them would provide us with new insight into the effects of diuron on stream biofilms. The previously demonstrated sensitivity of both methods (Kroll et al., 2014; Sgier et al., 2016) and diversity of endpoints gained from them lead also to the hypothesis that they could be more sensitive than the traditional endpoints. In the results and discussion, we compare the sensitivity and information content of the measured endpoints and their complementarity regarding temporal dynamics of diuron-related effects to stream biofilms.

## Materials and Methods

### Chemicals

Diuron used for the exposure studies was purchased from Sigma-Aldrich (Buchs, Switzerland) (DCMU, CAS 330-54-1, D2425), as were also the analytical grade diuron (DCMU, 45463) and Ammonium formate (10 M, 78314-100 ML-F) which were used for LC-MS measurements (see “Materials and Methods–Quantification of Diuron by LC-MS”). Acetonitrile HPLC gradient-grade purity was obtained from Acros Organics (Thermo Fisher Scientific, Reinach, Switzerland) and nanopure water from Barnstead NANOpure (Skan, Allschwil, Switzerland). All other chemicals were purchased from Sigma-Aldrich or Merck.

### Colonization of Natural Biofilms

Natural biofilms were colonized on glass microscope slides (38 mm × 26 mm, Thermo Fisher Scientific), which were placed vertically in Plexiglas channels in a flow-through system fed by water from the river Chriesbach (on campus, Dübendorf, Switzerland) (Navarro et al., 2007; Kroll et al., 2014). An overview of Chriesbach water chemistry is provided in Supplementary Table S1. A sediment trap (0.51 m × 0.7 m × 2.6 m, residence time about 20 min) was used to remove part of the dispersed particles. The flow rate in the channels was maintained at about 1 cm/s corresponding to a volume of 3 L/min. Illumination was provided in 12:12 h light/dark cycles by BioSun fluorescent tubes with a radiation similar to the sunlight spectrum (Radium Lampenwerk GmbH, Germany, ML-T8 36W/965/G13B). Temperature and photosynthetic active radiation (PAR) in the channels were monitored by a HOBO Pendant^®^Temperature/Light Data Loggers (UA-002-64) (median water temperature: 13.4°C [11.7–16.1°C], median light intensity in the light period: 1011.8 Lux).

### Diuron Exposure of Natural Biofilms in Indoor Microcosms

After 3 weeks of biofilm colonization, colonized glass slides were randomly transferred to 10 (Nos. 1–10) independent recirculating microcosms (Plexiglas, 10 cm × 25 cm, 3.5 cm water column, 32 glass slides per microcosm) containing 5 L of LA medium (Supplementary Table S2). Room temperature was maintained between 14.0 and 16.1°C resulting in median water temperature of 17.75°C (17.7–17.8°C). Light conditions and flow rate at the inflow were the same as during colonization (median light intensity in the light period: 947.2 Lux). Oxygen saturation and temperature were monitored in one microcosm (No. 10) with a Presens Microx TX3 system and an NTH-PSt1-L2.5-TS-NS40/0.8-YOP-EXT1 oxygen microsensor over 3 weeks (minimum [O_2_]/dark period: 7 mg/L, maximum [O_2_]/light period: 7.9 mg/L) and measured after 21 days in all microcosms. Conductivity, pH, and water chemistry were determined in parallel to sampling (Supplementary Tables S3, S4). One day after translocation, microcosms Nos. 1, 3, 5, 7, and 9 were exposed to 20 μg/L diuron, dissolved in methanol (CAS 330-54-1) during 3 weeks, whereas microcosms Nos. 2, 4, 6, 8, and 10 were exposed to methanol as solvent control [53.6 μL methanol added to 5 L of medium, equal to 0.001% (v/v) or 8.5 μg/L or 0.0008% (w/v)]. After each sampling time point at 7 (d_7_), 14 (d_14_), and 21 (d_21_) days after translocation, 3 L of the total 5 L LA medium was renewed to assure constant levels of nutrients and diuron (see “Materials and Methods–Quantification of Diuron by LC-MS”). As one of the main objectives of this study was to compare the effects of diuron on several different endpoints, the target diuron concentration was chosen close to the EC50 value reported in previous studies of diuron exposure, conducted on stream biofilms and individual freshwater algae species (European Service Innovation Scoreboard, 2000; Legrand et al., 2006; Knauer et al., 2007; Roubeix et al., 2011; Moisset et al., 2014; Kim Tiam et al., 2015).

### Sampling of Natural Biofilms From Microcosms

Immediately after translocation to the indoor microcosms (d_0_), and after 7 (d_7_), 14 (d_14_), and 21 (d_21_) days, 8 slides were taken from each microcosm and biofilms were pooled into 40 mL of LA medium with a plastic scraper. The suspension was used for FC-CS, taxonomy, biomass, chlorophyll-a content, photosynthetic efficiency analysis and EPS measurements. Of this 40 mL suspension, 10 mL were sonicated (45 kHz 60 W, VWR Ultrasonic Cleaner) for 1 min to break up colonies and immediately fixed with 0.01% paraformaldehyde and 0.1% glutaraldehyde (w/v, stock in tap water) and stored at 4°C for FC and taxonomic LM analysis. Further, 2 mL of the suspension were used for photosynthetic efficiency measurements and chlorophyll-a content measurements (PHYTO-PAM) and 8 ml were taken for biomass analysis. The generated supernatant was used for EPS extraction.

### Taxonomic Analysis by Light Microscopy

Fixed samples were diluted 1:10 three times independently in tap water and 1 mL was transferred to an Uthermol’s chamber for microscopic analysis. An inverted microscope (Zeiss Axiovert 135) was used to identify and count (Zeiss EC Plan-Neofluar 40×/0.75 objective, Zeiss EC Plan-Neofluar 100× 1.3 Oil objective, if necessary) algae and cyanobacteria genera and if possible species within three fields of vision, based on two taxonomic references and the recommendations by the Swiss Federal Office for the Environment (Hustedt, 1930; Pascher et al., 1987/1997; Hürlimann and Niederhauser, 2007). Counting was not possible for cells with colonial or filamentous growth. At least 150 cells were identified for each replicate.

### Biomass

To assess the biomass of the sampled biofilm, 8 mL of biofilm suspension of each sample were centrifuged at 2000 *g* for 10 min at room temperature and the resulting pellet was placed in a 2 ml Eppendorf tube and stored at -20°C for ∼72 h. Subsequently, the pellets were freeze-dried (LYOVAC GT2) for 24 h and dry weight measured.

### Photosynthetic Efficiency and Total Chlorophyll-a Content

Directly after sampling, photosynthetic efficiency was assessed by measuring the quantum yield of the photosystem II (ΦPSII) of 2 mL biofilm suspensions by Pulse-Amplitude-Modulated fluorometry (PHYTO-PAM, Walz Heinz GmbH) (Schreiber, 1998). In parallel to the photosynthetic efficiency measurements, the initial fluorescence (at 665 nm) was measured as an indirect measure of total chlorophyll-a content, using a constant sensitivity of the photomultiplier (gain) (Corcoll et al., 2011).

### EPS Extraction and Characterization

Extracellular polymeric substances were extracted from samples on d_0_, d_7_, d_14_, and d_21_ and were analyzed for organic carbon (OC) and organic nitrogen (ON) size distribution and protein content. The extraction procedure was performed as described previously (Stewart et al., 2013; Kroll et al., 2014). The supernatants generated by the biomass extraction were sequentially filtered using 1 μm glass fiber [VWR], 0.45 μm polypropylene [PALL], and 0.22 μm PES [Millipore] filters. Filters were washed with nanopure water (18.1 MΩ cm, Milli-Q) prior to use. EPS extracts were stored in glass bottles at 4°C [0.02% (w/v) NaN_3_]. All extraction steps were performed on ice, the water bath for ultrasound treatment was at room temperature.

Organic carbon and ON size distribution was measured by size-exclusion chromatography – organic carbon detection – organic nitrogen detection (LC-OCD-OND). Samples were diluted with nanopure water (18.1 MΩ cm, Milli-Q) directly before analysis. A size exclusion column (250 mm × 20 mm, Toyopearl TSK HW-50S) was used to separate EPS compounds. To quantify the carbon background of the extraction protocol, an aliquot of extraction buffer was treated the same way as periphyton suspensions and then assessed by LC-OCD-OND. The mobile phase was phosphate buffer (24 mM, pH 6.6) and the acidification solution was phosphoric acid (60 mM, pH 1.2). The detection limit was 10 μg/L for both OC and ON. The software FIFFIKUS was used to quantify total organic carbon (TOC), dissolved organic carbon (DOC), and chromatographable DOC compounds (cDOC). The chromatograms obtained from LC-OCD-OND are integrated to determine the amount of biopolymers (high M_r_ polysaccharides and proteins), building blocks of humic substances, low M_r_ acids, and amphiphilic/neutral compounds (alcohols, aldehydes, amino acids, and ketones).

Total protein in EPS extracts was measured by the Bradford assay using Bradford reagent (Bio-Rad Protein Assay Kit I) and an Infinite 200 (Tecan) plate reader. Calibration curves were produced with bovine serum albumin (BSA) diluted in equal amounts of EPS extracts to account for any interference of the EPS with protein detection.

### Community Structure Analysis by Flow Cytometry and viSNE

For single cell analysis of the biofilm communities, dichroic splitters and filters of the Beckmann Coulter Gallios flow cytometer (using 405, 488, 638 nm lasers) were selected to cover the fluorescence emission form 425–755 nm as previously described (Sgier et al., 2016). In total, 12 parameters were measured: forward (FS) and side scatter (SS), and 10 fluorescences (further explained in Supplementary Table S5). Before analyzing the biofilm suspension, the samples where filtered through 50 μm filters (CellTrics filter, Partec), as this is the particle size limit allowed by the used flow-cytometer. Three technical replicates per sample were measured. A total of 10,000 events per sample were acquired and gated to exclude all events that were above the signal saturation limit in any of the 12 parameters (<<1%). The area signal intensity of all parameters was exported as a csv-file and visualized with stochastic neighbor embedding (SNE) using the cyt software (Amir el et al., 2013). In short, SNE is a non-linear dimensionality reduction technique that is used for embedding high-dimensional data into a low-dimensional space (van der Maaten and Hinton, 2008). In our case, each measured cell was represented in 12-dimensions (FS, SS, 10 fluorescences) and SNE was used to embed the cells into a 2D space and then visualize the embedding using a scatter plot, i.e., viSNE map. Prior to visualization, technical and biological replicates were merged and random sampling was performed, so that the viSNE map was created from a mixture of equal-sized samples (18,000 cells/sample). Based on the visual clustering, subpopulations of the viSNE map were identified and the number of particles/cells that belonged to each subpopulation per sample was quantified. Subpopulations are defined as visually separable clusters of cells in the viSNE map, with optical and fluorescence properties that differentiate them from the other clusters. To facilitate the interpretation of the viSNE maps and the defined subpopulation, FC data of reference species (16 diatoms, 8 green algae, 6 cyanobacteria, and 1 red algae species, Supplementary Table S6) were projected on the viSNE map. For a more detailed description of the procedure, see (Sgier et al., 2016, 2018).

### Quantification of Diuron by LC-MS

The diuron concentration in the exposure media was measured every day during the first week of exposure, using LC-MS, to ensure a constant exposure level between two time points of media exchange. Ten microliters of sample were separated with the use of an Accela 600 HPLC pump with an in-line degasser (Thermo Fisher Scientific, San Jose, CA, United States), an HTS PAL auto sampler (CTC Analytics, Zwingen, Switzerland), which kept the samples at 4°C, and an Agilent Poroshell 120 EC, C18 column (2.1 mm × 100 mm, 2.7 μm particle size, Agilent Technologies AG, Switzerland). Pre-mixed eluents were used for the gradient (200 μl/min): A (90% Ammonium formate 5 mM in nanopure water and 10% Acetonitrile) and B (10% Ammonium formate 5 mM in nanopure water and 90% Acetonitrile). Starting conditions were 90% A with a linear gradient after 5.5 min to 60% A, followed by a linear gradient to 100% B in another 5.5 min, which was then kept for 1 min. Initial conditions were reached in 0.5 min and the column re-equilibrated for another 3 min. Before and after each injection, the syringe and auto sampler valve were cleaned once with 90% nanopure water/10% methanol and 100% methanol.

For mass spectrometry, the column was coupled to the Heated Electro Spray ESI inlet of a TSQ Vantage triple quad MS (Thermo Fisher Scientific, San Jose, CA, United States). The collision energy (CE) for the collision induced dissociation was optimized for the precursor (m/z = 233.1). ESI positive conditions were: needle voltage 4000 V, interface and vaporizer temperatures 250°C resp. 150°C, sheath gas 40, ion sweep gas 0.0 and aux gas 10. Q1 FWHM and Q3 FWHM were set to 0.7 resolution. Used scan width was 1.0 m/z and the scan time 0.1 s. Three transitions were monitored in single reaction monitoring (SRM) mode during the whole chromatogram. The MRM 233.1 – 72.1 (CE = 20) was used as analytical signal and the MRMs 233.1 – 46.1 (CE = 13), and 235.1 – 72.1 (CE = 19) as conformational signals. Peak areas were calculated by Thermo XCalibur^TM^ 3.0.63 software and manually adjusted before transferring to Excel, together with the retention times, for further processing. No sorption of diuron to the biomass was detected.

### Statistical Data Analysis

Because one of the main objectives of the study was to compare the different ecotoxicological endpoints, we decided to use only a few different statistical methods and apply them consistently with the measured endpoints.

For biomass, photosynthetic efficiency, chlorophyll-a content, individual FC fluorescences and individual clusters obtained from the viSNE analysis, we used linear modeling to analyze the effects of time and diuron exposure. For each endpoint, the linear model was fitted with maximum likelihood to model the respective endpoint as the dependent variable, and time and diuron exposure as fixed effects (with and without interactions). To determine whether time and treatment had an effect on any measured endpoint, we used the Two-way ANOVA and Tukey’s HSD test (Null hypothesis that neither time nor treatment have any effect on the endpoint). For extracted EPS comparison between d_0_ and d_21_, the *t*-test was used.

For taxonomy and FC-CS, we evaluated the effects of time and diuron exposure using permutational multivariate ANOVA based on dissimilarities (adonis), using R package *vegan* (jaccard distance was used as measure of dissimilarity, 10,000 permutations were used in all cases) (Dixon, 2003). All datasets were transformed using the Hellinger transformation before running adonis.

## Results

Biomass, chlorophyll-a content, photosynthetic efficiency, taxonomic composition, FC-CS and EPS were measured in long-term exposure experiments to diuron. To ensure a constant exposure concentration, diuron was quantified daily in each treated channel during the first week by LC-MS. Daily means were relatively stable (mean 20.22 ± SE 1.053 μg/L, Supplementary Figure S1), indicating that diuron was constant during the entire experiment.

Our hypothesis was that during the experiments the biofilms would undergo dynamic changes, which would include normal biofilm dynamics, the effects of translocation of the biofilms into the recirculating microcosm environment and the effects of exposure to diuron. We wanted to know how sensitive the different endpoints are to these factors (time, diuron treatment) and, for each respective endpoint, whether it is possible to detect the effects of both time and treatment or would one of them be dominant.

### Dynamics of Traditional Endpoints: Biomass, Photosynthetic Efficiency, Chlorophyll-a Content, Taxonomic Composition

**FIGURE 1:**
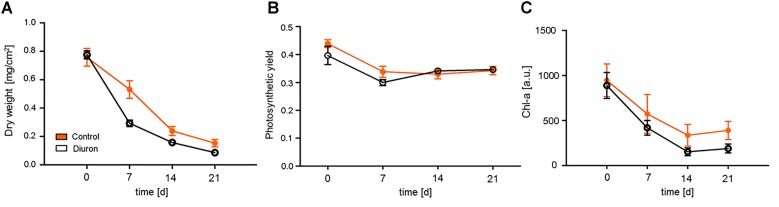
Measurement of traditional endpoints after diuron exposure. Changes between the control and diuron-treated community over time were measured as **(A)** dry weight, **(B)** photosynthetic efficiency (quantum yield of photosystem II) at 520 nm, and **(C)** chlorophyll-a content. For each time point, the data are given as mean ± SE, *n* = 5.

At the start of the experiment (d_0_), the control and exposed biofilms did not differ in biomass (measured as dry weight), photosynthetic efficiency or chlorophyll-a content (Figure 1). For both, exposed and control communities, time was the dominant factor, with all three endpoints decreasing over the course of the experiment [time effect for biomass: *F*_(3,36)_ = 78.25, *p* < 0.001; photosynthetic efficiency: *F*_(3,32)_ = 17.22, *p* < 0.001; chlorophyll-a content: *F*_(3,36)_ = 42.84, *p* < 0.001], while diuron exposure was not significant for any endpoint. For all endpoints, communities exposed to diuron showed a faster decrease within the first week of exposure, however, this was only significant for biomass after 1 week of exposure [time-treatment interaction: *F*_(3,32)_ = 4.23, *p* = 0.0125].

The overall decrease in biomass over the course of the experiment was also apparent in the genus-level taxonomic analysis (Supplementary Figure S2). In both, the control and the exposed communities, almost all detected genera decreased in abundance during the first week and stabilized towards the end of the experiment. The decrease in the diuron-treated communities was generally stronger, except for the genera *Diatoma* and *Gomphonema*, which increased in abundance (Supplementary Figure S2). The genus *Cocconeis* increased in abundance over the whole experiment in both control and diuron-treated communities. Nevertheless, permutational ANOVA of the taxonomic data did not find a significant time or treatment effect.

### Community Structure Analysis by Flow Cytometry and viSNE

For the FC part of the datasets, we decided to analyze the raw FC data (optical scatter and fluorescence intensities measured by the FC) and the FC data after categorization of the biofilms into subpopulations (FC-CS), separately. For the raw data, using permutational ANOVA we found the effects of time (*p* < 0.001) and diuron treatment (*p* = 0.0061) to be significant. Similar as for the traditional endpoints, the largest effect to measured optical and fluorescence properties was seen at 1 week after beginning of exposure. However, not all optical and scatter properties followed the same pattern; the effect of time was found to be significant only for forward scatter, side scatter (measures of size and granularity) and fluorescences FL 5–10 (see Supplementary Figure S3 for details), while the diuron effect was not statistically significant for any of the individual optical or scatter properties.

**FIGURE 2:**
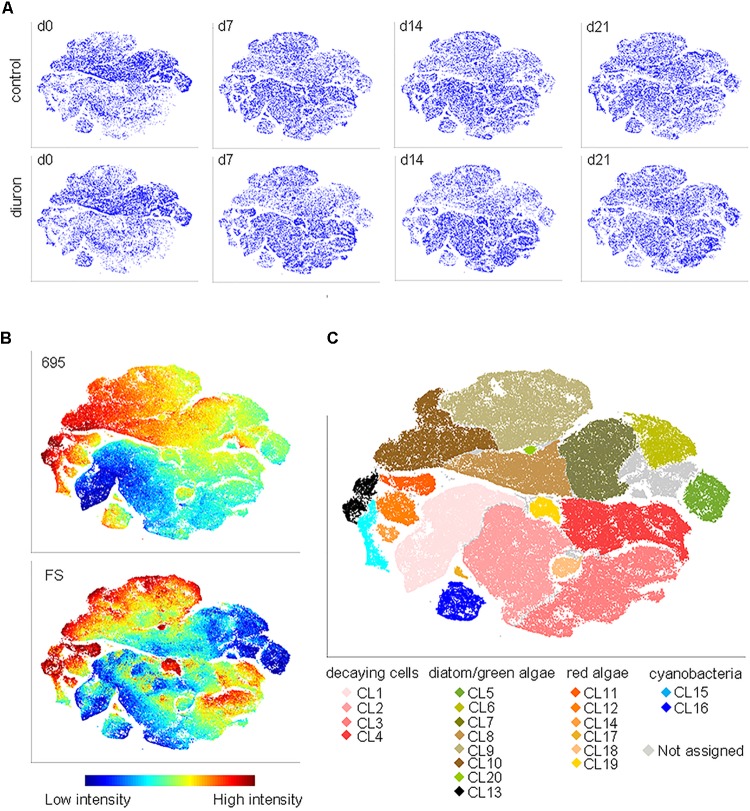
FC-CS in diuron-treated stream biofilms. **(A)** Biofilms were assessed by flow cytometry after sampling on d_0_, d_7_, d_14_, and d_21_, and altogether mapped by viSNE. viSNE maps are shown in single color, with each point in the viSNE map representing a single cell or particle from the biofilms or **(B)** colored according to fluorescence intensity at 695 nm and according to the forward scatter (full set of scattering and fluorescence intensities displayed in Supplementary Figure S4). **(C)** Subpopulations (SP 1–20) categorized based on the viSNE map and optical scatter and fluorescence intensities. Some cells (4.7%) were not assigned due to lack of distinct properties. Comparison of subpopulation properties with data acquired from reference species and pigment-bleached reference samples (Supplementary Figure S5) allowed for assigning subpopulations to types of organisms and potentially decaying cells.

**FIGURE 3:**
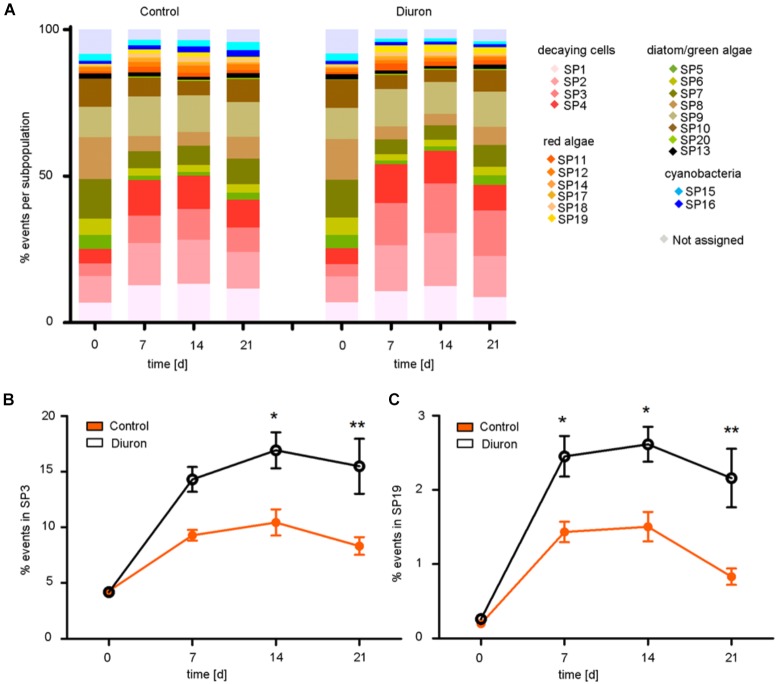
Tracking and quantification of FC-CS after diuron exposure. **(A)** Relative contribution of the subpopulation (%) to the community based on the viSNE map over time. Significant differences between control and diuron-treated community appeared in **(B)** subpopulation 3 after the second week and in **(C)** subpopulation 19 already after the first week, represented with ^∗^ and ^∗∗^ (ANOVA and Tukey HSD test for α = 0.05, ^∗^*P* ≤ 0.05, ^∗∗^*P* ≤ 0.01). Bars represent the standard error, *n* = 5.

Based on the viSNE map, we categorized 20 subpopulations (SP 1–20) according to their scattering and fluorescent properties (Figure 2 and Supplementary Figures S4, S5). The categorized subpopulations were further grouped into diatom-, green algae-, cyanobacteria-, red algae-, and decaying-like cells (Figure 2C), according to their specific fluorescence patterns (Supplementary Figure S4) and after projection of the reference species on the viSNE map (Supplementary Figure S6). Based on the relative contributions of the subpopulations to the communities, a strong shift in community structure in both, control and diuron-treated samples was apparent within the first week of the experiment (Figure 3A). Permutational ANOVA of the whole dataset found effects of both time (*p* < 0.001) and diuron treatment (*p* = 0.0035).

At the level of individual subpopulations, the most prominent changes were an increase in decaying-like cells (SP 1–4) and of cells similar to a subpopulation of the red algae *Bangia* (SP 19) on the one hand, and a decrease in most subpopulations similar to green algae and diatoms (SP 5, 6, 7, 8, 10) on the other hand (Figure 3A). Subpopulations similar to cyanobacteria remained relatively stable (SP 15, 16). Compared to the control community, diuron-treated communities showed a significantly stronger increase in decaying-like cells (SP 3) within the last 2 weeks [time-treatment interaction: *F*_(3,32)_ = 3.738 *p* = 0.02074, Tukey HSD at d_14_: *p* = 0.01655, d_21_: *p* = 0.00377] and an increase in red algae-like cells (SP 19) during the last 2 weeks of the exposure [time-treatment interaction: *F*_(3,32)_ = 2.9454, *p* = 0.04768, Tukey HSD at d_14_: *p* = 0.03628 d_21_: *p* = 0.00154] (Figures 3B,C). While the other subpopulations also showed differences between diuron and control, they were not significant (Supplementary Figure S7). The subpopulation of the red algae *Bangia* (SP 19) contained particularly large cells (higher forward and sideward scattering intensities) (Figures 2B,C).

### EPS Composition and Protein Concentration

**FIGURE 4:**
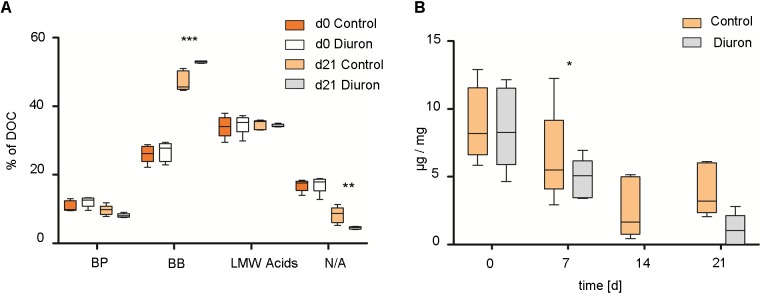
Extracellular polymeric substances (EPS) extraction of stream biofilm samples after diuron exposure. **(A)** EPS composition as % of total DOC after 3 weeks. BP, biopolymers; BB, building blocks of humic substances; LMW acids, low molecular weight acids; N/A, neutral/amphiphilic compounds. **(B)** Protein concentration per extracted DOC (μg/mg) over time. Data is presented as box plots according to Tukey. ^∗^, ^∗∗^, and ^∗∗∗^ represent the significant difference between the control and diuron-treated community at d21 (^∗^*P* ≤ 0.05, ^∗∗^*P* ≤ 0.01, ^∗∗∗^*P* ≤ 0.001), *n* = 5.

Total EPS extractable from biofilms increased from 4.63 μg EPS/mg dry weight/cm^2^ (range 3.42–5.84) at d_0_ to 9.72 μg EPS/mg dry weight/cm^2^ (range 7.44–12.22) at d_21_ in control communities and to 14.58 μg EPS/mg dry weight/cm^2^ (range 9.22–19.34) at d_21_ in diuron-treated communities. The means of total extractable EPS from control/diuron-treated communities on d_21_ were significantly different (*p* = 0.0348, *t* = 2.538, df = 8). Similarly, EPS composition was not significantly different between control/diuron-treated communities at the start of the experiment regarding the fraction of biopolymers (BP), building blocks (BB), low molecular weight acids (LMW acids), and neutral/amphiphilic compounds (N/A). They contributed by 14.98% BP (range 13.14–20.63), 33.42% BB (range 29.36–37.03), 35.86% LMW (range 32.66–38.78), and 13.73% N/A (range 11.46–15.36) to the total EPS. Within 3 weeks, the fraction of BP generally decreased, while that of BB increased (Figure 4A). The variability of biological replicates remained the same in control communities but substantially decreased in diuron-treated communities. Further, the fraction of BP and LMW acids was not significantly different between control and treated samples, while a significantly higher amount of BB and a lower amount of N/A was detected in EPS extracted from diuron-treated communities [treatment-component interaction: *F*_(3,32)_ = 17.694, *p* < 0.001, Tukey HSD: *p*(BB) < 0.001, *p*(N/A) = 0.009].

Protein concentration (μg protein/mg DOC) was slightly lower in EPS of diuron-treated communities after 1 week [treatment effect: *F*_(1,32)_ = 6.7107, *p* = 0.014], and continuously decreased in both, diuron and control communities [time effect: *F*_(3,32)_ = 22.0321, *p* < 0.001]. It was below detection limit in EPS extracted from diuron-treated communities at d_14_ (Figure 4B), but recovered in 3 out of 5 treated communities.

## Discussion

The objective of this study was to evaluate the effects of long-term (3-week) exposure of stream biofilms to diuron and compare the sensitivity and information content among endpoints traditionally used in biofilm characterization (biomass, photosynthetic efficiency, chlorophyll-a content, taxonomic composition) and some recently developed endpoints (FC-CS and EPS content). Specifically, we measured the biofilm dynamics under control and diuron exposure conditions and asked whether it is possible to detect time and exposure-related effects by using the different endpoints.

### Endpoint Sensitivity

All measured endpoints were strongly affected by the dynamics of the biofilm communities, independent of the treatment. In particular, we observed temporal variation in abundance of most genera, in biomass, in cell size (as given by FC forward scatter) and the presence of decaying cells (as measured by FC), in photosynthetic efficiency (PAM) and in the EPS composition. This expected dynamics can be explained by the continuous changes of the complex community composition of stream biofilms and has been observed in previous studies with a similar experimental setup (Ricart et al., 2009; Tlili et al., 2011; Kim Tiam et al., 2015). A time-related effect was statistically detected by all endpoints used, except for genus-level taxonomic composition of the biofilms. More than indicating a lack of sensitivity of the method, the lack of detection of a time effect in this case probably lies in the relatively low number of cells taxonomically identified.

A diuron effect was only detected by the FC community structure analysis and by the analysis of EPS, while the other endpoints were only indicative of an exposure effect. For example, during the experiment the dry weight, chlorophyll-a content, photosynthetic efficiency and the abundances of several identified genera decreased more in the diuron exposed biofilms, but the difference was not statistically significant. Since the diuron concentration used in the experiments was relatively high, a lack of detection of the diuron effects on some endpoints was rather surprising, considering previous research in biofilms and in single species (Dorigo et al., 2007; Pesce et al., 2010b; Moisset et al., 2014). However, temperature and other co-varying environmental factors have been shown to mask the effects of chemicals exposure in biofilms (Pesce et al., 2010b; Romero et al., 2018), and in our case the masking effect was apparently large enough to prevent detection of a diuron effect by several endpoints. As our biofilms were colonized using local stream water, another potential explanation is that the communities were tolerant to diuron because of its presence in the water. While we have not measured diuron in the stream water, we find this explanation unlikely as the experiments were performed in winter when the use of herbicides is very low.

There have been several studies before that used complementary endpoints to characterize effects of stressors on biofilms, and depending on the stressor and endpoint used, the sensitivity of different endpoints was reported differently. In other diuron studies, Kim Tiam et al. (2015) found that photosynthetic efficiency was a more sensitive indicator of stress than chlorophyll-a content for stream biofilms (1–33 μg/L; 13 day exposure), Moisset et al. (2014) found photosynthetic efficiency more sensitive than cell density for three species of diatoms (10 μg/L; 10 day exposure) Ricart et al. (2009) found biovolume to be more sensitive than photosynthetic efficiency and chlorophyll-a content (5 μg/L; 30 day exposure). This suggests that even in relatively comparable experimental setups, with the same chemical stressor, it is difficult to predict which endpoint is the most sensitive. While our results do not match each of the previous studies, they do confirm that traditional endpoints are not always sensitive enough to detect changes in stream biofilms after stress.

### Endpoint Complementarity

Since the endpoints used in the study measure different features of the biofilm, it was expected that they will point at different aspects of biofilm dynamics. FC and EPS both allowed the detection of diuron-exposure related effects. While using single-cell optical and fluorescent properties directly already enabled this detection, the data were difficult to interpret. The interpretation was made easier after subcategorization of the cells in different taxonomic and phenotypic groups, i.e., diuron exposure significantly increased the number of decaying and dying cells in the samples (compared to controls) and also significantly increased the abundance of red algae with large cells, while green algae and diatoms were only somewhat reduced in abundance. The relatively small reduction in green-algae and diatoms are further corroborated by the (not statistically significant) decrease in chlorophyll-a content and biomass in diuron exposed biofilms, while the increase in red algae were qualitatively confirmed using light microscopy (the growth form of *Bangia* prevented quantification). At this time, it is worth mentioning that generally the dynamics of different taxonomic groups during the experiment were the same regardless if measured by FC or microscopy. The communities studied here were apparently able to compensate diuron-induced effects on photosynthetic efficiency, which indicates that there is functional redundancy, explainable by the compensatory effects of the species fluctuation in the community (Micheli et al., 1999; Valdivia et al., 2012).

Analysis of the EPS composition revealed a decrease in the ratio of protein concentration to extracted DOC in diuron-treated communities, a measure of the C:N ratio in the extracted DOC. The chemical composition of EPS depends among others on species composition, including both, phototrophic and heterotrophic organisms. The described outcome is in line with the changes in species abundance discussed above. Similarly, application of 20 μg/L of ionic silver triggered an increase of the C/N or DOC to protein ratio in EPS in stream biofilms (Kroll et al., 2016).

We suggest that the increase in C/N or DOC to protein ratio observed in the present study could be due to (glycol) proteins in EPS being produced in larger amounts by the algal community which is affected by diuron, while application of ionic silver, which is commonly used as bactericide, mostly affected the heterotrophic part of the community. The reduced heterotrophic community would therefore produce less polysaccharides resulting in a higher C/N ratio. Interestingly, the diuron treatment substantially decreased the variability of the EPS composition in diuron-treated communities. The variability in concentration of EPS components in the diuron-treated samples was substantially lower than in the control samples, indicating that there may be a response to diuron on the biofilm level which leads to a very tightly regulated EPS composition. Finally, although not statistically significant, the taxonomic analysis suggests that the diatom genera *Gomphonema*, *Diatoma*, and *Cocconeis* have tolerated the diuron exposure better than other genera, with *Gomphonema* and *Diatoma* even increasing in abundance. Our previous work has shown that all these genera overlap in fluorescence properties with other diatom genera; therefore it would not be possible to detect their advantage in diuron exposure with FC.

### Limitations of the New Methods

Although this, and our previous studies have demonstrated the potential of the new methods, more studies are needed to see whether they work equally well in a variety of environmental conditions. We have so far shown that FC-CS can be a good indicator of changes in biofilms under temperature (Sgier et al., 2016) and chemical stress, and that it can be evaluated in a relatively short amount of time once set up (Sgier et al., 2018). However, missing are studies that would measure FC-CS across a gradient of chemical concentration of different chemicals to see what the limits of sensitivity are. Also, the reference algal FC library that we have used for interpretation of the results is only available for conditions similar as in our laboratory set up. It needs to be investigated to which extent our library would be equally useful for interpretation of biofilm samples taken from other freshwater systems or whether a system-specific libraries would have to be established.

The extracellular polymeric matrix of the biofilms, consists of a complex mixture of EPS produced by the cells present in the biofilm (Battin et al., 2016). The composition of the EPS, which is usually dominated by proteins and polysaccharides, depends on the type of microorganisms present, but also on the age of the biofilm and the environmental conditions the biofilm is exposed to (Pan et al., 2016). The ratio of sugars and proteins in the biofilm is suggested to be a good indicator of environmental change, however, measuring both requires use of different methods and the measurements are not yet standardized. The interpretation of the results beyond simple detection of change is also challenging, as the intracellular EPS-synthesis are not yet completely understood. Simultaneous measurement of EPS and global metaOmics (e.g., metagenomics, metatranscriptomics) has the potential to connect the measured EPS changes with their molecular causes and thus enable a more mechanistic interpretation of the results.

## Conclusion

The specific results obtained from different endpoints in this study confirms that a combination of endpoints and monitoring over a certain period of time are necessary to understand long-term effects of a stressor on stream biofilms (Micheli et al., 1999). One important reason is that the functional traditional endpoints are directly connected to the ecological function of the biofilm in the stream and therefore have direct functional relevance, which is not (yet) true for the new endpoints. We have shown that multiple measurements, such as in the case of red algae abundance and phenotype as measured by light microscopy and FC, can increase the confidence of the results and improve their interpretation. However, this study has also shown that the new tested endpoints, FC-CS and EPS composition, can, at least under certain conditions, be more sensitive than the traditionally used ones and therefore should be further tested in scientific and regulatory biofilm characterization and monitoring.

## Author Contributions

LS planned and conducted the study and wrote the manuscript. RB advised the study and contributed to the manuscript. RS performed the LC-MS measurements and contributed to the manuscript. AZ contributed to data analyses and wrote the manuscript. AK conceived, planned, and conducted the study and wrote the manuscript.

## Conflict of Interest Statement

The authors declare that the research was conducted in the absence of any commercial or financial relationships that could be construed as a potential conflict of interest.
