# Supplementary material for: Evaluation of Phototrophic Stream Biofilms Under Stress: Comparing Traditional and Novel Ecotoxicological Endpoints After Exposure to Diuron
Source: Front Microbiol. 2018 Nov 29;9:2974. doi: 10.3389/fmicb.2018.02974 (PMC6281688; doi:10.3389/fmicb.2018.02974)
Supplement: Supplementary file 1 [file Data_Sheet_1.PDF]

Supplementary Information

Supplementary Figures

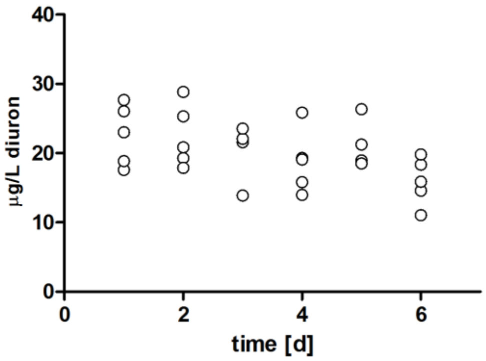

Figure S1. Diuron stability . Diuron concentration [μg/L] in the LA media over time as measured by LC-MS. Data is presented as individual measurements.

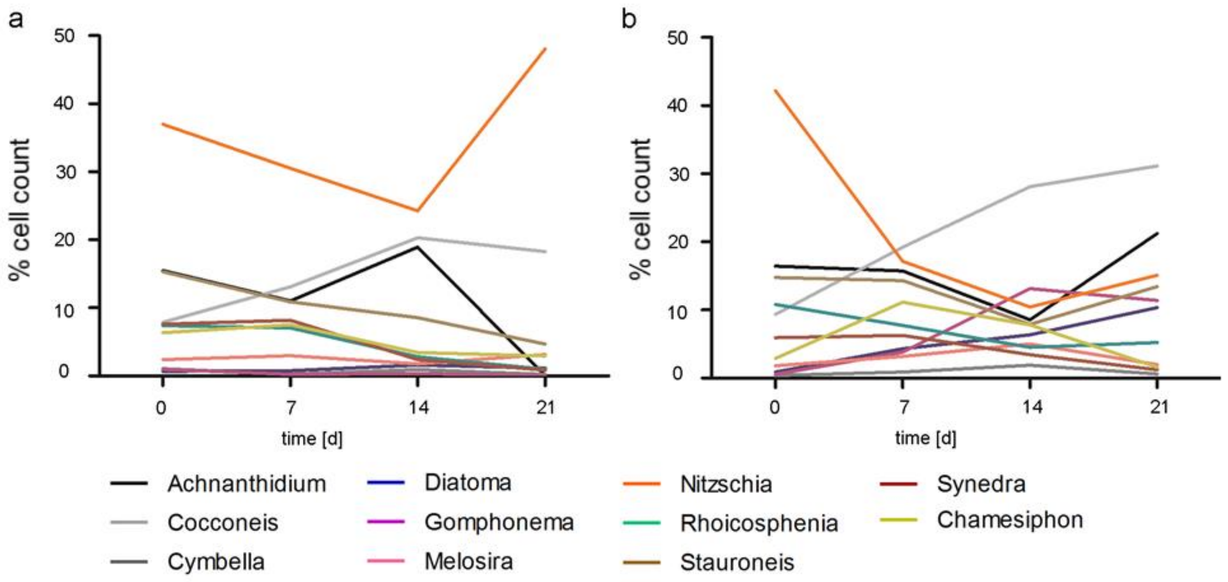

Figure S2. **Cell counts of detected genera in the stream biofilm after diuron exposure.** Number of cells per genus per cm^2 identified **a)** in control communities and **b)** in the diuron-treated community. Data is presented as % average cell counts per total cell counts per microcosm per time point.

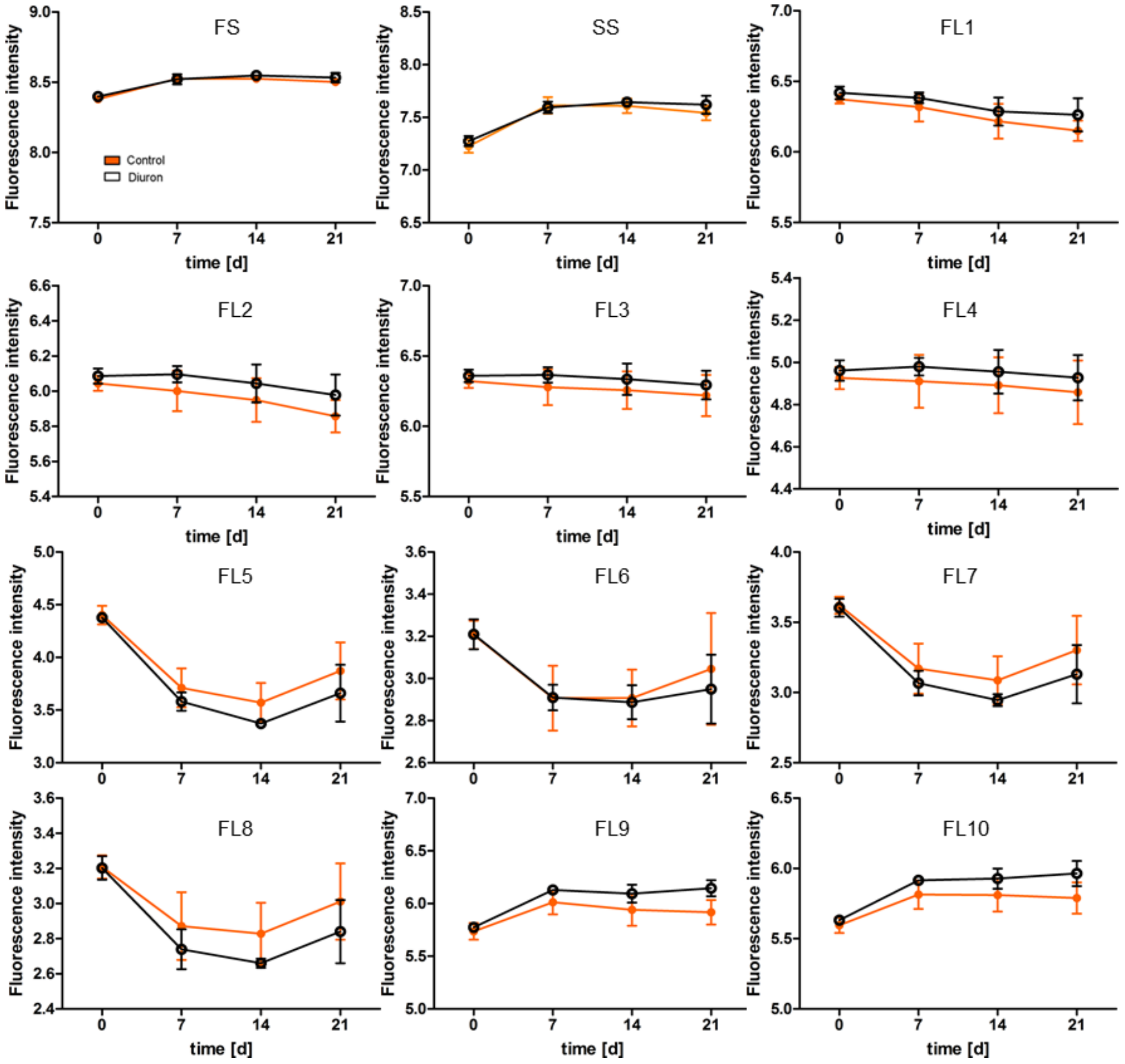

Figure S3. Optical and fluorescent properties of the biofilm. Optical scatter (forward scatter FS and sideward scatter SS) and fluorescence intensity at specific wavelengths [nm] measured by flow-cytometry; given as mean ± SE (n = 5). Time effect significant for FS ($F(3,36) = 56.23$, $p<0.001$); SS ($F(3,36) = 73.89$, $p<0.001$); FL5 ($F(3,36) = 65.45$, $p<0.001$); FL6 ($F(3,36) = 64.12$, $p<0.001$); FL7 ($F(3,36) = 63.45$, $p<0.001$); FL8 ($F(3,36) = 62.86$, $p<0.001$); FL9 ($F(3,36) = 35.56$, $p<0.001$); FL10 ($F(3,36) = 32.11$, $p<0.001$).

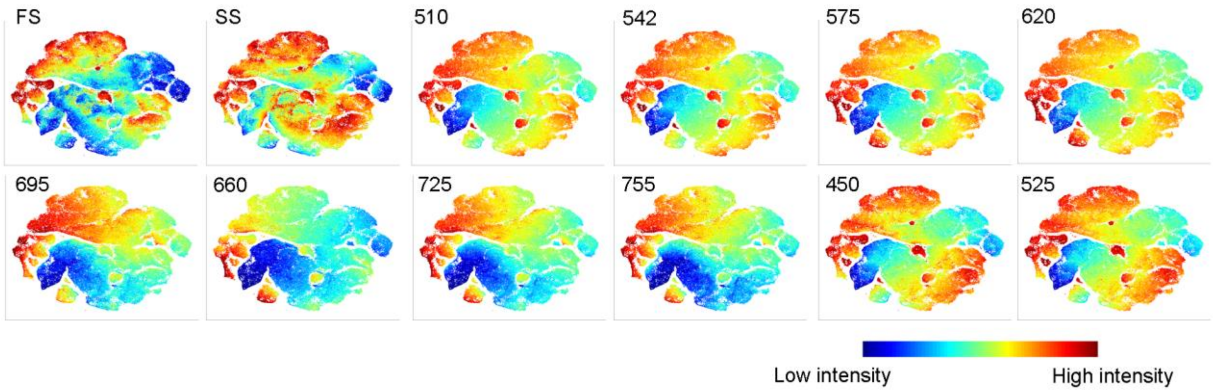

Figure S4. Coloured viSNE map from Figure 3. ViSNE maps are colored according to optical scatter (forward scatter FS and sideward scatter SS) and fluorescence intensity at specific wavelengths [nm] measured by flow-cytometry.

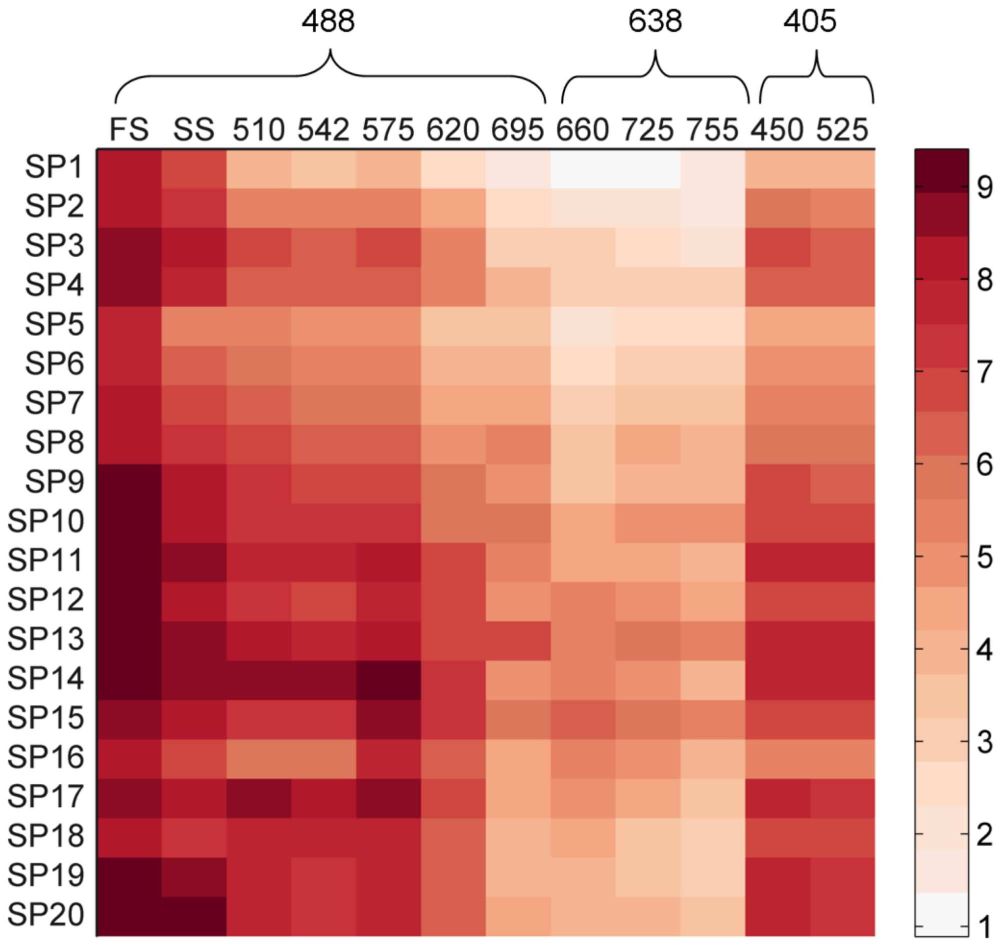

Figure S5. Heatmap of optical scatter and fluorescence intensities of each subpopulation defined in Figure 3c. Laser wavelength [nm] (above the) and filter wavelength [nm] are shown at top of the figure.

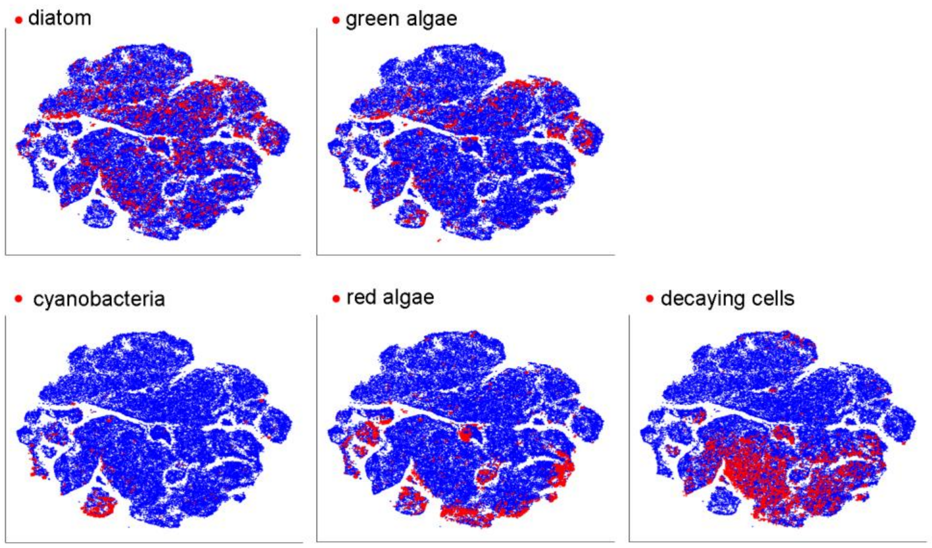

Figure S6. Projection of flow cytometry data points taken from reference species. Reference species are grouped into diatoms, green algae, cyanobacteria, red algae as well as pigment-bleached (decaying cells) reference samples onto the viSNE map shown in Fig 3a. Additional information about the reference species can be found in Table S6.

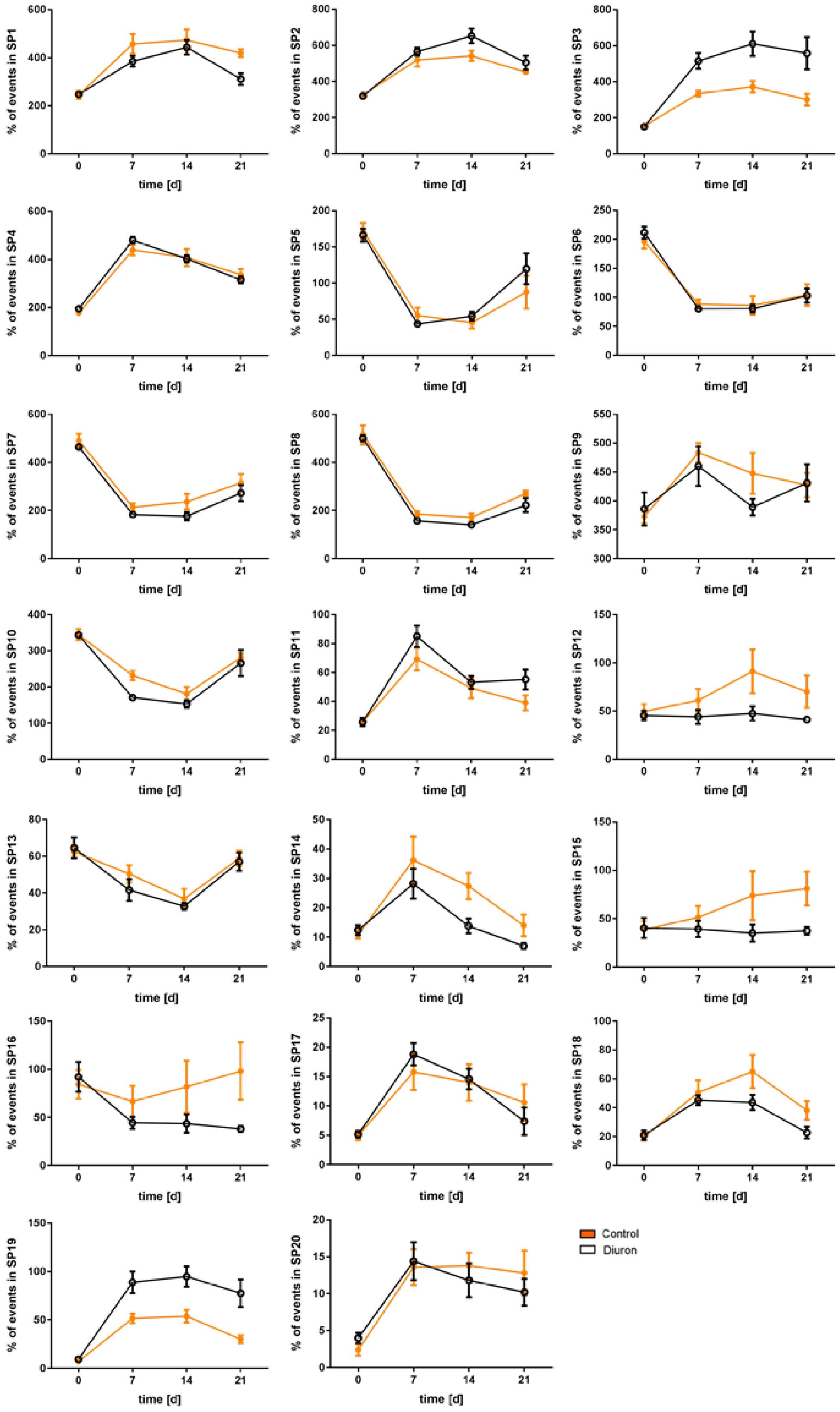

Figure S7. Subpopulations identified in the biofilms (from Figure 2). Number of events in each subpopulation identified by FC; given as mean ± SE (n = 5). All subpopulations had a significant time effect ($p < 0.001$), while only SP3 and SP19 were significant also for a diuron effect (see Figure 3).

Supplementary Tables

Table S1. Water chemistry of River Chriesbach. The table lists values determined in this study in water samples taken when biofilms where sampled for exposure (11/2014) and average values published by the Canton Zurich Office of Waste, Water, Energy and Air (WWEA) for 2014.

	**Chloride [mg/L]**	**Nitrate - N [mg N/L]**	**Sulfate [mg/L]**	**Na [mg/L]**	**Mg [mg/L]**	**Ca [mg/L]**	**K [mg/L]**	**o - P [µg/L]**	**D - P [µg/L]**	**T - P [µg/L]**	**H_4SiO_4 [mg/L]**	**DOC [mg/L]**	**TOC [mg/L]**
11/2014	28.7	4.9	20	19	3.1	101.8	13.9	53.7	56.8	84.3	11.9	2.7	2.7
averages in 2014*		6.29							116	141		2.68	

*Canton Zurich Office of Waste, Water, Energy and Air (WWEA), http://www.hw.zh.ch/chemie/fg/177_L.pdf.

Table S2. Medium LA composition given in [mM]. Components 1-9 are mixed and autoclaved, components 10-17 are mixed and sterile-filtered (0.22 µm).

	Component	Concentration [mM]
1	$Ca(NO_3)_2$ $4H_2O$	0.1
2	$MgSO_4$ $7H_2O$	0.15
3	$NaHCO_3$	6.2
4	K_2HPO_4 $3H_2O$	0.005
5	NH_4NO_3	0.1
6	$NaNO_3$	0.1
7	Na_2SiO_3 $5H_2O$	0.05
8	$CaCl_2$ $2H_2O$	0.25
9	KNO_3	0.1
10	H_3BO_3	0.05
11	$ZnSO_4$ $7H_2O$	0.000158
12	$MnCl_2$ $4H_2O$	0.00122
13	$CoCl_2$ $6H_20$	0.00005
14	$CuSO_4$	0.000163
15	Na_2MoO_4 $2H_2O$	0.00008
16	$FeCl_3$ $6H_2O$	0.0009
17	Na_2 EDTA	0.02

Table S3. Water chemistry. Water chemistry as measured on d 0, d 7, d 14, and d 21 of the experiment; M: Microcosm.

	M	Chloride [mg/L]	Nitrate - N [mg N/L]	Sulfate [mg/L]	Na [mg/L]	Mg [mg/L]	Ca [mg/L]	K [mg/L]	o - P [µg/L]	D - P [µg/L]	T - P [µg/L]	H_4SiO_4 [mg/L]	DOC [mg/L]	TOC [mg/L]
d_0	all	26.3	6.2	13	125	3.6	13.7	4.1	134	147	212	3.7	9.3	10.6
d_1	1	26.3	7.1	13	147	3.7	13.4	4.6	18.3	32.6	69.5	4.9	5.6	6.6
	2	28.8	7.3	13	143	3.7	13.6	6.2	13.4	25.1	60.4	5.1	5.2	6.2
	3	16.9	9.5	12	134	3.7	12.6	4.3	2.3	15.2	28.3	<0.5	4.8	5.0
	4	25.8	7.2	12	140	3.6	13.5	4.4	8.5	19.1	41.2	5.3	4.7	5.3
	5	17.3	9.7	13	138	3.7	12.7	4.4	1.5	13.3	38.1	<0.5	5.4	5.4
	6	27.4	7.8	13	152	3.8	13.8	4.2	23.0	33.8	46.4	4.6	4.9	5.2
	7	26.0	7.8	13	140	3.9	13.7	4.3	14.4	28.1	57.5	3.4	4.9	5.4
	8	26.2	7.4	13	141	3.7	13.5	4.3	8.6	18.6	31.1	4.3	4.6	5.7
	9	25.0	7.8	13	143	3.8	13.6	4.4	7.5	18.7	42.4	<0.5	5.2	5.4
	10	30.7	7.4	13	146	3.8	13.6	7.1	6.3	18.8	36.6	5.0	4.4	4.5
d_{14}	1	29.6	7.2	13	150	3.7	10.8	8.9	7.4	20.8	42.7	7.2	5.3	5.3
	2	24.8	7.0	14	146	3.7	10.8	5.6	3.4	12.5	25.3	8.0	4.7	5.2
	3	12.7	9.7	13	145	3.7	10.4	4.5	1.6	9.5	26.3	<0.5	4.5	4.8
	4	23.6	7.0	14	143	3.7	10.9	4.6	1.9	11.8	22.0	12.8	4.5	4.7
	5	19.5	8.2	13	153	3.7	10.5	4.4	1.3	10.4	34.0	<0.5	4.5	5.5
	6	23.5	7.3	14	146	3.9	11.0	4.6	2.7	12.5	26.5	6.1	4.3	4.7
	7	16.2	8.3	13	152	3.8	10.7	5.3	3.3	11.8	37.3	3.5	4.4	5.0
	8	22.5	6.8	13	143	3.7	10.6	5.4	1.1	9.4	26.9	6.1	4.0	4.5
	9	22.2	7.1	13	150	3.7	10.7	5.2	2.2	10.4	27.7	<0.5	4.6	4.6
	10	24.8	6.9	13	153	3.8	10.9	6.1	1.1	15.6	39.4	7.4	4.5	4.8
d_{21}	1	31.8	5.9	14	149	3.6	12.5	9.1	2.7	11.6	47.0	7.0	4.6	4.6
	2	35.5	6.7	13	241	3.5	7.7	5.0	3.2	12.6	51.0	6.9	4.3	4.7
	3	23.7	7.5	13	139	3.6	11.9	4.5	1.2	8.9	30.9	<0.5	4.2	4.4
	4	29.3	6.8	14	153	3.8	12.9	4.8	1.3	8.6	24.9	1.0	4.6	4.6
	5	26.4	7.5	14	146	3.7	12.5	4.5	0.5	9.5	72.0	<0.5	4.3	4.9
	6	29.2	6.8	14	136	3.8	13.1	4.9	1.3	10.0	24.7	<0.5	4.1	4.3
	7	23.6	7.1	13	137	3.6	12.2	4.3	1.9	10.0	16.3	<0.5	4.2	4.8
	8	27.2	6.7	13	145	3.7	12.4	4.5	1.4	8.1	19.5	3.0	4.0	4.6
	9	27.0	7.1	13	146	3.7	12.7	5.1	1.7	11.4	43.7	<0.5	4.4	4.5
	10	29.0	6.6	14	147	3.8	12.7	5.1	1.9	9.9	22.4	7.4	4.4	4.6

Table S4. Oxygen concentration. Oxygen concentration 10 h after the start of the light period after 21 d; electrical conductivity (CD) and pH 4 h after the start of the light period on d 1, d 7, d 14, and d 21 of the experiment.

Microcosm	O_2 [mg/L]	Electrical conductivity [mS]				pH			
		d 1	d 7	d 14	d 21	d 1	d 7	d 14	d 21
1	7.7	0.653	0.686	0.730	0.726	8.58	8.50	8.25	8.25
2	7.9	0.647	0.640	0.682	0.636	8.59	8.28	8.44	8.56
3	7.9	0.647	0.637	0.636	0.651	8.58	8.37	8.42	8.40
4	7.9	0.651	0.646	0.668	0.671	8.61	8.42	8.40	8.39
5	7.9	0.627	0.629	0.652	0.678	8.62	8.40	8.41	8.41
6	7.8	0.688	0.708	0.678	0.687	8.62	8.42	8.40	8.39
7	7.8	0.663	0.628	0.629	0.638	8.58	8.41	8.38	8.41
8	7.8	0.639	0.649	0.643	0.636	8.56	8.31	8.25	8.36
9	7.7	0.655	0.645	0.642	0.667	8.48	8.35	8.32	8.35
10	7.7	0.672	0.735	0.676	0.685	8.49	8.32	8.34	8.34

Table S5. Gallios flow cytometer hardware and software settings. *set to 1-19°

Parameter	Laser [nm]	Dicroic splitter	Filter/band width [nm]	Voltage	Gain
FS*,[+]	488	-	-	10	2
SS[++]	488	-	-	10	1
FL1	488	525	510/20	519	1
FL2	488	550	542/27	636	1
FL3	488	595	575/30	759	1
FL4	488	655	620/30	701	1
FL5	488	-	695/30	495	1
FL6	638	710	660/20	250	1
FL7	638	750	725/20	250	1
FL8	638	-	755/LP	250	1
FL9	405	480	450/50	386	1
FL10	405	-	525/40	347	1

[+] Forward scatter. When the laser light hits the cell, it scatters, depending on the ratio between size and laser wavelength. Forward scatter is measured by a detector positioned along the path of the laser light and allows for discrimination of cells by size (FS intensity is proportional to the diameter of the cell).

[++] Side scatter. Side scatter is measured at a ninety degree angle relative to the laser. It is generally of lower intensity than forward scatter and provides information about the internal granularity of the cell.

Table S6. Algal and cyanobacterial reference strains. Strains were obtained from Experimental Phycology and Culture Collection of Algae at the University of Goettingen (EPSAG), Culture Collection of Algae at the University of Cologne (CCAC), Thonon Culture Collection (TCC), and University of Texas Culture Collection of Algae (UTEX), or were available at Eawag.

Genus	Species	Bank	Strain number
Achnanthes	*sp.*	CCAC	CCAC 2681 B
Achnanthidium	*minutissimum*	TCC	TCC746
Anabaena	*sp.*	Eawag isolate	
Bangia	*atropurpurea*	EPSAG	1351-1
Botryococcus	*braunii*	CCAC	CCAC 0121
Chamaesiphon	*polonicus*	EPSAG	32.87
Chlorella	*sp.*	Eawag isolate	
Cocconeis	*placentula var. Euglypta*	TCC	TCC720
Craticula	*accomoda*	TCC	TCC107
Cyclotella	*meneghiniana*	EPSAG	1020-1a
Cymbella	*sp.*	CCAC	CCAC 2680 B
Diatoma	*sp.*	CCAC	CCAC 3717 B
Eolimna	*minima*	TCC	TCC524
Fragilaria	*perminuta*	TCC	TCC882
Gomphonema	*parvulum*	TCC	TCC653
Gomphonema	*parvulum*	EPSAG	1032-1
Merismopedia	*glauca*	EPSAG	48.79
Microcystis	*aeruginosa*	unknown	PCC 7806
Mougotia	*sp.*	EPSAG	11.96
Nitzschia	*palea*	TCC	TCC139-2
Nitzschia	*palea*	EPSAG	1052-3a
Oedogonium	*sp.*	EPSAG	54.94
Phormidium	*sp.*	Eawag isolate	
Pseudanabaena	*galeata*	EPSAG	13.83
Scenedesmus	*acuminatus*	EPSAG	38.81
Scenedesmus	*bimorphus*	Eawag isolate	
Stigeoclonium	*aestivale*	EPSAG	477-20
Surirella	*sp.*	CCAC	CCAC 3461 B
Ulnaria	*ulna*	TCC	TCC634
Ulothrix	*mucosa*	EPSAG	56.9
